# The impact of social deprivation on reverse total shoulder arthroplasty outcomes

**DOI:** 10.1016/j.xrrt.2025.100589

**Published:** 2025-10-07

**Authors:** Kelly E. Jacoby, Logan M. Andryk, Andrew Valiquette, Matthew Van Boxtel, Evan Cox, Steven I. Grindel, Alexander Graf

**Affiliations:** Department of Orthopaedic Surgery, Medical College of Wisconsin, Milwaukee, WI, USA

**Keywords:** Reverse shoulder arthroplasty, Social deprivation, Area deprivation index, Rotator cuff arthropathy, Patient-reported outcomes, Orthopedic surgery, Health disparities, Socioeconomic status

## Abstract

**Background:**

Social deprivation has previously been shown to have a negative correlation with patients' overall health. In addition, higher levels of social deprivation have been shown to be correlated with poor postoperative outcomes following orthopedic procedures and health-care–related quality of life. The purpose of this study is to evaluate how a patient's level of social deprivation level, as measured by the Area Deprivation Index (ADI), affects patients with rotator cuff arthropathy (RCA) and their experience with reverse total shoulder arthroplasty (rTSA).

**Methods:**

A retrospective review, with an evidence level of 3, composed of 119 patients (133 shoulders) with primary RCA who underwent rTSA by a single surgeon at a single institution from 2005-2020. Social deprivation was determined using the patient's ADI score. Preoperative and postoperative range of motion, visual analog scale scores, American Shoulder and Elbow Surgeon scores, Constant–Murley Scores, and Simple Shoulder Test scores were recorded for each patient. Patients were grouped into terciles based on their level of social deprivation and comparisons were made between the groups. Analysis of variance and student t-testing were used to determine statistically significant differences between the groups.

**Results:**

Significant functional improvements were observed following rTSA for patients with RCA. Preoperatively, patients from the most deprived group showed lower average Simple Shoulder Test scores (1.62 vs. 3.04, *P* = .026), while postoperatively, these patients showed lower external rotation with their arm at the side (33° vs. 42°, *P* = .044). Otherwise, there were no significant differences in pain or functional outcomes between the ADI groups preoperatively or postoperatively. Notching and postoperative complication rates were also not statistically different between the 3 groups (*P* = .886 and *P* = .697, respectively).

**Conclusion:**

rTSA is a safe and effective procedure for patients from all levels of social deprivation, and patients can experience similar postoperative shoulder pain and function regardless of their socioeconomic status.

The incidence of reverse total shoulder arthroplasty (rTSA) procedures continues to increase in popularity and prevalence in the United States.^4^ In fact, the growth rate of rTSA is now outpacing that of hip and knee arthroplasty.^4^ This is secondary to expanding surgical indications and improvements in implant design, surgeon training, and clinical outcomes over time.^6^ However, the role of patient social factors on clinical outcomes remains less understood with some studies demonstrating equivalent outcomes,^9^ and increased rates of readmission and complications in patients of socioeconomic disadvantage.^3^

Understanding the impact of social determinants of health (SDOH) on rTSA outcomes is important for developing comprehensive and patient-centered approaches to orthopedic care. Historically, social deprivation has been used as an SDOH metric that reflects patients' access to health resources and socioeconomic status. It has been shown to negatively correlate with patient outcomes after orthopedic procedures.^10^ More recently, the Area Deprivation Index (ADI) has emerged to measure social deprivation as it relates to a patient's specific geography. Therefore, this study assesses the impact of social deprivation, as measured by the ADI, on clinical outcomes following rTSA. We hypothesize that patients with higher levels of social deprivation will experience less improvement in postoperative outcomes such as pain, range of motion (ROM), and patient-reported outcomes (PROs), and that they will have higher complication rates.

## Materials and methods

A retrospective cohort study was conducted, comprising 119 patients (133 shoulders) who underwent primary rTSA for a diagnosis of rotator cuff arthropathy (RCA). The study cohort had a mean age of 70.3 ± 9.0 years and consisted of 82 female and 37 male patients. All procedures were performed by a single surgeon at a single academic institution between January 2005 and May 2020. Patients were identified usingCurrent Procedural Terminology (CPT) code 23472 within the institutional database.

Inclusion criteria consisted of patients aged 18 years or older with a diagnosis of primary RCA, who had complete preoperative and postoperative clinical data and a minimum of 6 months of clinical follow-up. Exclusion criteria included revision rTSA, incomplete clinical documentation, and insufficient follow-up duration (<6 months).

Social deprivation was assessed using the ADI. ADI scores were obtained using the publicly available Neighborhood Atlas developed by the University of Wisconsin School of Medicine and Public Health. This is a validated geographic-based composite score derived from 17 census variables related to income, education, housing quality, and employment. ADI scores range from 1 to 100, with higher scores indicating greater deprivation.^16^ Scores were calculated based on the patient's residential address and categorized into terciles: least deprived (0-33rd percentile), moderately deprived (34-67th percentile), and most deprived (68-100th percentile).

Preoperative and postoperative data were collected for ROM, visual analog scale (VAS) pain scores, American Shoulder and Elbow Surgeons (ASES) scores, Constant–Murley Scores, and Simple Shoulder Test (SST) scores. ROM measurements were obtained through direct physical examination by the operating surgeon or the physician assistant during preoperative and postoperative clinical visits. Complication data were obtained through comprehensive chart and radiographic review and included scapular notching, intraoperative fracture, postoperative acromial stress fracture, infection, dislocation, nerve injury, implant failure, and the need for revision surgery.

Statistical comparisons between ADI groups were performed using 1-way analysis of variance for continuous variables and Student's t-tests for pairwise comparisons. A *P* value of <0.05 was considered statistically significant.

## Results

### Study population

A total of 133 patients met the inclusion criteria for this study. Patients were stratified into ADI terciles: the least deprived group included 33 patients, the moderately deprived group included 56 patients, and the most deprived group included 44 patients.

### Preoperative differences

Both preoperative and postoperative patient-reported outcome measures were analyzed. At baseline, patients in the most deprived tercile demonstrated significantly lower SST scores compared to those in the least and moderately deprived groups (*P* = .026 and *P* = .004, respectively). Additionally, the most deprived group had significantly higher preoperative VAS pain scores than the moderately deprived group (*P* = .024), though the difference was not statistically significant when compared to the least deprived group (*P* = .502). Preoperative external rotation with the arm at the side was also reduced in the most deprived group compared to the least deprived group (*P* = .050).

### Postoperative pain outcomes

Despite these baseline differences, postoperative outcomes were largely similar across ADI groups. A statistically significant difference persisted in external rotation at the side between the most and least deprived groups (*P* = .044), although this difference was small and not clinically meaningful. No other patient-reported outcome measures or ROM outcomes demonstrated significant variation postoperatively between terciles.

**Figure 1:**
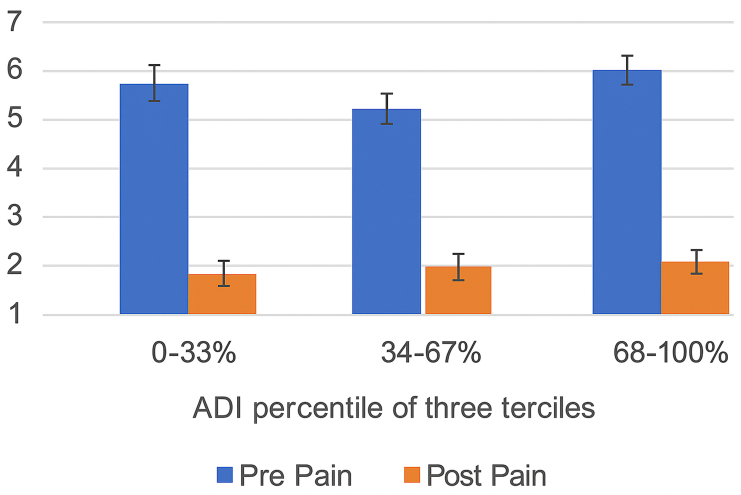
ADI tercile vs. pain score. *ADI*, Area Deprivation Index.

VAS pain scores improved in all 3 groups. Patients in the least deprived tercile improved from 6.27 preoperatively to 1.07 postoperatively. The moderately deprived group improved from 5.50 to 1.51, and the most deprived group improved from 6.68 to 1.95. These improvements are illustrated in Figure 1.

### Postoperative ROM

ROM improved in all terciles. Forward flexion increased from 88.94° to 136.67° in the least deprived group, 81.67° to 136.09° in the moderately deprived group, and 77.67° to 130.81° in the most deprived group.

External rotation at the side improved from 39.55° to 42.19°, 30.56° to 35.64°, and 28.95° to 33.26°, respectively.

External rotation at 90° increased from 49.38° to 62.83°, 36.10° to 60.10°, and 43.82° to 61.22° in the respective groups.

**Figure 2:**
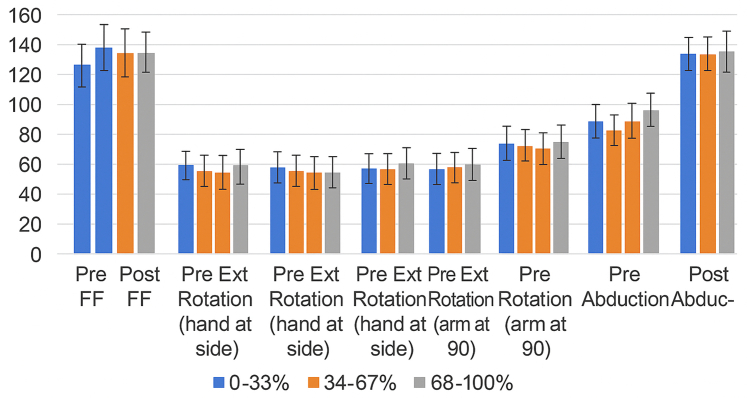
ADI tercile vs. range of motion. *ADI*, Area Deprivation Index.

Abduction increased from 78.94° to 126.21° in the least deprived group, 78.68° to 125.00° in the moderately deprived group, and 69.42° to 122.02° in the most deprived group. These ROM improvements are summarized in Figure 2.

### Postoperative PROs

Constant–Murley Scores increased postoperatively from 32.33 to 69.35, 33.00 to 66.84, and 27.72 to 64.26 across the least, moderate, and most deprived groups, respectively.

SST scores improved from 3.04 to 8.38, 3.02 to 7.90, and 1.62 to 6.97, respectively.

**Figure 3:**
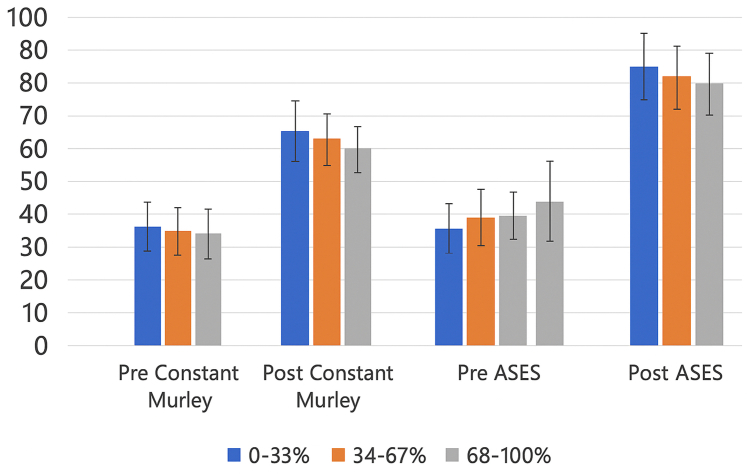
ADI tercile vs. Constant–Murley and ASES scores. *ASES*, American Shoulder and Elbow Surgeons; *ADI*, Area Deprivation Index.

**Figure 4:**
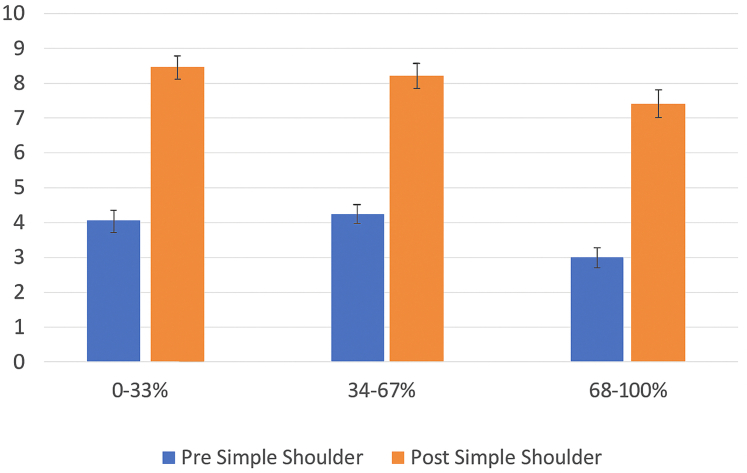
ADI tercile vs. simple shoulder test scores. *ADI*, Area Deprivation Index.

Similarly, ASES scores increased from 36.56 to 81.73, 41.37 to 78.92, and 34.32 to 74.36. These findings are illustrated in Figures 3 and 4.

## Complications

Complication rates, including the presence of scapular notching, did not significantly differ between ADI groups (*P* = .886 and *P* = .697, respectively). A summary of all outcome comparisons between terciles is provided in Table I, with statistically significant differences denoted.

**Table I:** Outcome differences by ADI tercile.

Comparing 2 terciles (*P* values)	Least deprived (1) vs. moderately deprived (2)	Least deprived (1) vs. most deprived (3)	Moderately deprived (2) vs. most deprived (3)
Visual analog score preop	0.147	0.502	0.024∗
Visual analog score postop	0.393	0.163	0.424
Forward flexion preop	0.444	0.233	0.607
Forward flexion postop	0.907	0.27	0.296
External rotation (at-side) preop	0.099	0.05∗	0.728
External rotation (at-side) postop	0.078	0.044∗	0.499
External rotation (at 90) preop	0.055	0.465	0.229
External rotation (at 90) postop	0.562	0.767	0.818
Abduction preop	0.974	0.236	0.174
Abduction postop	0.832	0.497	0.611
Constant–Murley Preop	0.849	0.204	0.129
Constant–Murley Postop	0.399	0.162	0.461
ASES Preop	0.195	0.609	0.058
ASES Postop	0.512	0.173	0.396
Simple shoulder test preop	0.981	0.026∗	0.004∗
Simple shoulder test postop	0.488	0.069	0.197

*ADI*, Area Deprivation Index; *ASES*, American Shoulder and Elbow Surgeons.

∗ Asterisk indicates statistically significant differences (*P* < .05).

## Discussion

Social determinants of health have been shown to affect patient outcomes following orthopedic procedures.^10^ Several prior studies have utilized ADI as a more accurate marker for social deprivation and true understanding of social determinants of health. ADI can represent a more accurate depiction of a patient's local environment as it relates to access to care and other barriers.^12^

The only variable that demonstrated statistically significant difference between groups was external rotation with the arm at the side between the most and least deprived terciles. Studies evaluating shoulder motion after arthroplasty suggest that a minimum clinically important difference (MCID) for external rotation improvements may be as low as 2°.^11^ In our study, the least deprived group gained an average of 2.6° in external rotation postoperatively, while the most deprived group gained 3.3°. The magnitude of change falls within the expected MCID threshold, suggesting that this subtle difference is unlikely to reach clinical relevance.^11^^,^^15^ Additionally, pain relief and patient-reported outcomes often correlate more strongly with satisfaction than minor changes in ROM.^11^

Despite worse preoperative function and pain in patients from the most deprived tercile, we found that all patients, regardless of social deprivation, achieved similar outcomes after rTSA. Recent literature regarding social deprivation and shoulder surgeries are mixed. Our results are consistent with another study of 380 patients that reported no significant differences in postoperative PROs (ASES, Single Assessment Numeric Evaluation, VAS) for those undergoing elective shoulder arthroplasty, regardless of whether they lived in more disadvantaged neighborhoods, as measured by ADI.^9^

A 2024 study utilizing Medicare claims data and ADI demonstrated higher 90-day readmission rates as greater health-care contact days in the first 90 days as compared with the lowest ADI group. Our study did not include 90-day readmission rates or health-care utilization rates.^2^ However, in a prior study, our institution did not find a significant difference in hospital resource utilization (clinic, emergency department, or readmission) in the least deprived ADI tercile. This may represent the smaller cohort of 780 patients included in this prior study or may represent a regional difference compared to the general Medicare data.^14^

A 2025 multicenter study explored the relationship between ADI and clinical outcomes as well as implant survivorship, in a cohort of 1,148 patients (including both anatomic and reverse arthroplasty). They found a weak but negative correlation with national ADI and preoperative and postoperative motion, showing poorer functional outcomes based on PROs as well as diminished improvement following surgery. No differences were noted in revision free survival between the groups. Although they did note a mild statistical difference in outcomes, their results are quite similar to those of our cohort.^7^

Outside of the shoulder arthroplasty literature, a large systematic review of SDOH and rotator cuff repair performed recently included 32 studies and encompassed over 100,000 patients. They demonstrated variables including racial and ethnic minority status, low-income place of residence and low volume surgery regions, and unemployment as variables that contributed to a delay in access to care and/or more severe presentation.^8^ Similarly, our results reflected a more advanced preoperative disease state in our patient population, as demonstrated by lower SST scores, higher VAS scores, and poorer ROM at presentation. In this systematic review, however, outcomes following rotator cuff repair were worse in these disadvantaged groups identified. Our study did not identify similar postoperative differences in clinical outcomes. This could be secondary to the prolonged rehabilitation and recovery process required following RCR vs. that of rTSA.

The literature also highlights disparities in surgical access related to social deprivation. A study by Testa and colleagues examined the relationship between social determinants of health and shoulder instability surgery, finding that patients from areas with higher social deprivation index scores (stratified by zip code) were less likely to undergo surgical intervention. Additionally, Hispanic, and Black patients had lower surgical rates compared to White patients, and individuals with Medicaid or self-pay insurance were less likely to receive surgery than those with private insurance.^13^ This study did not report on patient outcomes.

Overall, research suggests that while socioeconomic factors such as income and insurance type can impact access to care and complication rates, they may have less influence on long-term outcomes like functional recovery.^1^ Our results and those of other similar studies also speak to the durability and rehabilitation potential of reverse shoulder arthroplasty.^5^ Many studies that have demonstrated differences in outcomes by ADI or other markers of SDOH have focused on procedures that require a significant amount of dedicated rehabilitation following surgery.

This study has several limitations inherent to its retrospective design. Although efforts were made to ensure consistency in surgical technique, rehabilitation protocols, follow-up schedules, and outcome measurement, certain factors may limit the generalizability of our findings. These include a relatively small sample size, variability in patient comorbidities, severity of RCA, and potential differences in adherence to physical therapy. Additionally, complications treated outside our health system may not have been captured.

Notably, the study did not include 90-day readmission rates or emergency department utilization data, which may reflect early postoperative complications or disparities in access to care. Furthermore, while we observed meaningful improvements in range of motion and patient-reported outcomes, we did not assess the proportion of patients who achieved MCID, substantial clinical difference, or patient-acceptable symptom state thresholds. These represent limitations of our outcome assessment and opportunities for future research.

## Conclusion

This study highlights that while patients from more socially deprived backgrounds exhibited worse preoperative pain and function, they experience similar postoperative outcomes following reverse shoulder arthroplasty when compared to their less deprived counterparts. This suggests that rTSA is a reliable procedure across varying levels of social deprivation, providing significant functional improvements regardless of socioeconomic factors. Future research should focus on larger cohorts and explore additional variables such as long-term outcomes, which could further enhance the understanding of the relationship between social determinants and orthopedic outcomes.

## Acknowledgment

The authors would like to thank Karen Gonzalez, Clinical Research Coordinator for the Department of Orthopaedic Surgery at the Medical College of Wisconsin, for her assistance in facilitating this research.

## Disclaimers:

Funding: No funding was disclosed by the authors.

Conflicts of interest: The authors, their immediate families, and any research foundations with which they are affiliated have not received any financial payments or other benefits from any commercial entity related to the subject of this article.

## References

[1] Alvarez P.M., McKeon J.F., Spitzer A.I. (2022). Socioeconomic factors affecting outcomes in total knee and hip arthroplasty: a systematic review on healthcare disparities. Arthroplasty.

[2] Bethell M.A., Mahoney H.R., Adu-Kwarteng K., Kiwinda L.V., Clark A.G., Hammill B.G. (2025). The impact of socioeconomic factors on 90-day postoperative readmissions and cost in shoulder arthroplasty patients. J Shoulder Elbow Surg.

[3] Farronato D.M., Pezzulo J.D., Rondon A.J., Sherman M.B., Davis D.E. (2023). Distressed communities demonstrate increased readmission and healthcare utilization following shoulder arthroplasty. J Shoulder Elbow Surg.

[4] Farley K.X., Wilson J.M., Kumar A., Gottschalk M.B., Daly C., Sanchez-Sotelo J. (2021). Prevalence of shoulder arthroplasty in the United States and the increasing burden of revision shoulder arthroplasty. JBJS Open Access.

[5] Goldfarb C.A., King J.J., Wright T.W., Schoch B.S., Farmer K.W., Wright J.O. (2024). Rehabilitation after reverse total shoulder arthroplasty: a survey of members of the American shoulder and elbow surgeons. J Shoulder Elbow Surg.

[6] Hermena S., Rednam M. (2024). StatPearls.

[7] Khlopas A., Wright L.T., Hao K.A., Reddy A., Beason A., Simcox T. (2025). The effect of socioeconomic status on clinical outcomes and implant survivorship after primary anatomic and reverse total shoulder arthroplasty. J Shoulder Elbow Surg.

[8] Mandalia K., Ames A., Parzick J.C., Ives K., Ross G., Shah S. (2023). Social determinants of health influence clinical outcomes of patients undergoing rotator cuff repair: a systematic review. J Shoulder Elbow Surg.

[9] Moverman M.A., Sudah S.Y., Puzzitiello R.N., Pagani N.R., Hart P.A., Swanson D. (2022). Neighborhood socioeconomic disadvantage does not predict outcomes or cost after elective shoulder arthroplasty. J Shoulder Elbow Surg.

[10] Sheth M.M., Morris B.J., Laughlin M.S., Elkousy H.A., Edwards T.B. (2020). Lower socioeconomic status is associated with worse preoperative function, pain, and increased opioid use in patients with primary glenohumeral osteoarthritis. J Am Acad Orthop Surg.

[11] Simovitch R.W., Flurin P.H., Wright T.W., Zuckerman J.D., Roche C.P., Grey S.G. (2018). Quantifying success after total shoulder arthroplasty: the minimal clinically important difference. J Shoulder Elbow Surg.

[12] Singh G.K. (2003). Area deprivation and widening inequalities in US mortality, 1969–1998. Am J Public Health.

[13] Testa E.J., Bozic J.A., Abboud J.A., Maltenfort M.G., Levin L.S., Dines J.S. (2022). Social and demographic factors impact shoulder stabilization surgery in anterior glenohumeral instability. J Shoulder Elbow Surg.

[14] Van Boxtel M.E., Jauregui I., Valiquette A., Sullivan C., Graf A., Hanley J. (2024). The effect of social deprivation on hospital utilization following distal radius fracture treatment. J Hand Surg Glob Online.

[15] Werner B.C., Chang B., Nguyen J.T., Dines D.M., Gulotta L.V. (2018). What change in American shoulder and elbow surgeons score represents a clinically significant change after shoulder arthroplasty?. Clin Orthop Relat Res.

[16] Kind A.J.H., Buckingham W.R. (2018). Making neighborhood-disadvantage metrics accessible: the neighborhood atlas. N Engl J Med.

